# Visual outcomes and quality of life after bilateral extended depth of field, bifocal, and mix-and-match IOL implantation

**DOI:** 10.1371/journal.pone.0341136

**Published:** 2026-02-06

**Authors:** Xiaobao Liu, Yabo Fu, Yue Huang, Yulong Huang, Zhi Lin, Qinchun Zheng, Wenjie Wu

**Affiliations:** 1 Shengli Clinical Medical College of Fujian Medical University, Fuzhou, China; 2 State Key Laboratory of Ophthalmology, Zhongshan Ophthalmic Center, Sun Yat-sen University, Guangdong, China; 3 Department of Ophthalmology, Longyan People Hospital of Fujian, Longyan, China; 4 Equipment Material Department, Fuzhou University Affiliated Provincial Hospital, Fuzhou, China; 5 Department of Ophthalmology, Fuzhou University Affiliated Provincial Hospital, Fuzhou, China; Democritus University of Thrace, GREECE

## Abstract

**Purpose:**

To evaluate the visual outcomes and vision-related quality of life among three intraocular lens (IOL) implantation strategies: bilateral extended depth of field (EDoF) IOL, bilateral bifocal IOL, and a mix-and-match approach.

**Methods:**

In this prospective, non-randomized, observational study, patients with bilateral age-related cataracts selected their preferred IOL implantation type and were categorized into three groups accordingly. Three months after surgery, assessments of corrected and uncorrected binocular visual acuity at far, intermediate, and near distances were conducted, along with refraction, defocus curve, stereoacuity, vision-related quality of life, and photic symptoms.

**Results:**

A total of 76 patients (152 eyes) were included. All patients achieved uncorrected distance visual acuity (UDVA) and best corrected distance visual acuity (BCDVA) of 20/32 or better in all groups. Binocular uncorrected intermediate visual acuity and distance-corrected intermediate visual acuity were superior in the mix-and-match and bilateral EDoF IOL groups (both P < 0.01). The bilateral bifocal IOL and mix-and-match groups demonstrated better distance-corrected intermediate visual acuity (both P < 0.05). No significant differences were observed among the three groups in UDVA, BCDVA, or uncorrected near visual acuity (UNVA) (P > 0.05). All groups reported similar outcomes for stereoacuity, vision-related quality of life, and photic symptoms (P > 0.05).

**Conclusions:**

All three IOL strategies provided favorable vision-related quality of life, with the mix-and-match and bilateral EDoF IOL groups showing superior intermediate vision. The mix-and-match and bilateral bifocal IOL groups demonstrated better distance-corrected bilateral near vision performance, despite comparable UNVA among the three groups. These approaches remain valuable alternatives for providing optimal visual outcomes in regions where full vision range IOL are not yet accessible.

## Introduction

Recent advancements in intraocular lens (IOL) technology have greatly expanded the options for cataract surgery, offering both patients and surgeons a wider array of choices. Premium IOLs, such as multifocal and extended depth of field (EDoF) IOLs, facilitate postoperative vision across multiple distances, potentially correcting presbyopia and reducing the need for spectacles after cataract surgery [1–3].

The TECNIS ZMB00 IOL is a bifocal, diffractive lens with a + 4.0 D near addition, commonly used in presbyopia-correcting cataract surgery. While it provides good near vision, particularly for Asians with its + 4.0 D near addition, its intermediate vision has been reported as less effective [4–6]. In contrast, the TECNIS Symfony ZXR00 is an EDoF IOL that offers consistent vision from distant to intermediate ranges but lacks near vision capability [7,8]. Currently, no single IOL type, including trifocal IOL or EDoF multifocal IOL, has yet achieved seamless and all-distance vision nor guaranteed complete spectacle independence postoperatively [6,7] While EDoF multifocal IOL and trifocal IOL come closer to achieving this goal, they remain largely inaccessible in many developing countries, such as China, and their cost is still prohibitive for a significant portion of cataract patients. Consequently, bifocal IOL and EDoF IOL remain widely used and popular premium IOLs in China.

In recent years, the widespread adoption of e-payment applications in China has made mobile phone usage indispensable, including among the individuals undergoing cataract surgery [9–12]. People now rely on their mobile phones for nearly all activities, including payments, shopping, transportation and entertainment. This shift may result in different visual needs for the Chinese cataract patients, especially concerning near vision, compared to both Western cataract populations and Chinese cataract patients from the previous decade. Furthermore, intermediate vision has also become increasingly important due to the growing engagement in various daily, occupational, and recreational activities that require intermediate visual focus. Theoretically, Full vision range IOL, such as trifocal IOL, may be well-suited to meet the evolving demands for near and intermediate vision [6,7]. However, in many regions of China, trifocal IOLs remain unavailable within the public healthcare system, on which more than 90% of the Chinese population relies [13]. Even in our medical center, one of the largest in Fujian Province, trifocal IOLs are still inaccessible, and bifocal and EDoF IOLs remain the mainstream. Consequently, a “mix-and-match approach”, defined as the implantation of an EDoF IOL in one eye and a bifocal IOL in the fellow eye, is often employed as a compromise surgical strategy [6,8,14–16].

In addition to objective visual performance, patient-reported visual function and quality of life have become essential outcome measures following cataract surgery. Vision-related quality of life is commonly assessed using validated questionnaires, which capture patients’ subjective visual experiences in daily activities. Among these instruments, the Chinese version of the Catquest-9SF questionnaire has been extensively validated and widely used to evaluate visual function and postoperative satisfaction in Chinese cataract populations, demonstrating good reliability, validity, and sensitivity to visual changes after intraocular lens implantation [17,18].

However, given the limited availability of IOL types and the growing demand for varied visual distances in China, it remains unclear whether bifocal and EDoF IOLs can adequately address these changing needs. Therefore, the aim of this study is to evaluate the visual outcomes and quality of life associated with different surgical strategies: bilateral EDoF IOLs, bilateral bifocal IOLs, and the mix-and-match approach.

## Methods

### Participants

In this prospective, non-randomized observational study, patients diagnosed with bilateral age-related cataracts at Fuzhou University Affiliated Provincial Hospital between October 2022 and October 2023, who were willing to undergo second-eye surgery within a one-week interval, were included. The study adhered to the principles of the Declaration of Helsinki and received approval from the Ethics Committee of Fuzhou University Affiliated Provincial Hospital (K2022-06–008). All patients were informed of potential benefits and risks and provided written consent.

The inclusion criteria were as follows: (1) diagnosis of bilateral age-related cataracts; (2) surgical interval between the two eyes within one week; (3) corneal astigmatism less than 0.75 D; (4) axial length between 22 mm and 26 mm; (5) willingness to participate and complete all follow-up visits. Patients with a history of trauma, uveitis, glaucoma, macular degeneration, cystoid macular edema, proliferative diabetic retinopathy, corneal dystrophy, keratoconus, significant irregular astigmatism, or other ocular pathologies potentially resulting in postoperative visual acuity worse than 0.2 logMAR were excluded.

Before enrollment and surgery, all patients underwent a comprehensive ophthalmological examination including objective and subjective refractions, uncorrected distance visual acuity (UDVA) and best corrected distance visual acuity (BCDVA), slit-lamp biomicroscopy, non-contact tonometry, optical biometry (IOL Master 700, Carl Zeiss Meditec, Jena, Germany), corneal topography (Pentacam HD, Oculus Optikgeräte GmbH, Wetzlar, Germany), and fundus evaluation. Then, the surgeon discussed with the participants their personal lifestyles and vision requirements by using a short questionnaire (S1 Appendix). Based on their own preferences, participants were categorized into three groups: those receiving bilateral EDoF IOL implantation (bilateral EDoF IOL group); those receiving bilateral bifocal IOL implantation (bilateral bifocal IOL group); and those receiving a mix-and-match approach (mix-and-match group), defined as implantation of an EDoF IOL in the dominant eye and a bifocal IOL in the nondominant eye.

### Surgery technique

All surgeries were performed by a single experienced surgeon (W.J.W.) using a standard phacoemulsification under topical anesthesia. A 2.4-mm clear corneal incision was made, followed by continuous curvilinear capsulorrhexis with a diameter of 5.5 mm, nucleus removal, cortical aspiration, and posterior capsular polishing. After refilling the capsular bag with an ophthalmic viscosurgical device (OVD), the IOL was inserted into the capsular bag. The procedure was then completed with OVD aspiration and a watertight closure of the surgical wounds.

### IOL design

Both IOL models used were posterior chamber, one-piece, soft-foldable acrylic, hydrophobic, UV-absorbing, diffractive lenses. Both lenses featured a biconvex optic with a wavefront-designed aspheric optic to compensate for corneal spherical aberration, along with a square posterior edge and a frosted optic edge design. The TECNIS Symfony ZXR00 (Johnson & Johnson Surgical Vision, Inc., Santa Ana, CA, USA) incorporated an achromatic diffractive pattern and unique echelette design, extending the depth of focus and compensating for corneal chromatic aberration. In contrast, the TECNIS ZMB00 (Johnson & Johnson Surgical Vision, Inc., Santa Ana, CA, USA) was a diffractive multifocal IOL with a + 4.00 D add power, lacking the echelette feature but distributing light into two focal points. Emmetropic IOL power was selected using the Barrett Universal II formula with keratometry, axial length, and anterior chamber depth measured by the IOL Master 700.

### Postoperative examination

The participants were scheduled to follow up at one week and one month and three months after surgery. During these visits, before the 3-month follow-up, UDVA, BCDVA, intraocular pressure, slit-lamp and fundus examination, as well as any adverse event were all recorded. At the 3-month postoperative visit, both corrected and uncorrected binocular visual acuity was assessed at far (5 m), intermediate (66 cm), and near (40 cm) distances. Additional assessments included manifest refraction, defocus curve, stereoacuity, vision-related quality of life, and photic symptoms.

Defocus curves were plotted by measuring visual acuity under photopic conditions at 5 m, with lenses added in 0.5 D increments from −4.0 to +1.0 D.

Vision-related quality of life was evaluated using the Chinese Catquest-9SF questionnaire, a Chinese-translated version that has been proven unidimensional and reliable for assessing visual function in Chinese cataract patients (Table 3) [17,18]. The questionnaire includes seven questions related to daily-life activities and two global questions concerning general difficulties and overall satisfaction with vision. Responses are rated on a scale of 1 = very great difficulty, 2 = great difficulty, 3 = some difficulty, and 4 = no difficulty. Lower scores indicate better visual function.

Photic symptoms were evaluated through a separate questionnaire, where participants were shown diagrams depicting glare and halo. Participants were asked to rate the severity of these photic phenomena in daily life on a scale from 0 (none) to 5 (very severe) (S2 Appendix).

Near stereoacuity at 0.4 m was measured using the Titmus stereo test under photopic conditions without near-vision correction. Stereoacuity was recorded based on the number of correctly identified circles and converted into seconds of arc (arc sec) for statistical analysis. A stereoacuity level corresponding to circle 5 (100 arc sec), were considered the lowest threshold for useful stereoacuity. The mean stereoacuity and number (percentage) of patients who achieved a disparity threshold of 100 or 40 arc sec or better, were compared across the three groups.

### Statistical analysis

Visual acuity values were transformed to logMAR notation for statistical analysis. Sample size estimation was performed prior to study initiation using PASS (v.15, NCSS, Kaysville, Utah, USA). According to the results of a previous study, the visual acuity of 0.18 logMAR with standard deviation of 0.23 logMAR was recognized as a minimal relevant mean difference [19]. Considering a dropout rate of about 20%, the sample size of 84 participants (n = 28 in each group) was targeted to achieve 90% statistical power to detect an α = 0.05 based on one-way analysis of variance contrasts. Data analysis was completed using the SPSS program (v. 24, SPSS Inc., Chicago, Illinois, USA). Results of descriptive analysis are presented as mean and standard deviation. Intergroup comparisons were performed using one-way analysis of variance, followed by Tukey’s post hoc analysis. Categorical variables were compared using the Fisher exact probability test or the chi-square test. P values less than 0.05 were considered statistically significant.

## Results

In this study, 76 patients (152 eyes) completed all follow-up visits and were included for final analysis. There were 34 (44.7%) males and 42 (55.3%) females; the mean age of participants was 69.82 years. Table 1 summarizes the characteristics of all participants. No intraoperative or postoperative complications were reported.

**Table 1:** Patient characteristics.

Characteristic	Mix-and-Match Group	Bilateral Bifocal IOL Group	Bilateral EDoF IOL Group	P Value
Participants/eyes (n)	26/52	28/54	22/44	
Male/Female (n)	11/15	14/14	9/13	
Age (years)	69.88 ± 7.14	69.36 ± 6.72	70.32 ± 7.05	0.78
Axial length (mm)	23.51 ± 0.79	23.34 ± 0.82	23.65 ± 0.57	0.13
Anterior chamber depth (mm)	3.47 ± 0.58	3.28 ± 0.55	3.09 ± 0.42	0.01*
Keratometry (D)	43.56 ± 1.18	43.62 ± 1.61	43.51 ± 1.29	0.93
Corneal astigmatism (D)	−0.39 ± 0.18	−0.37 ± 0.17	0.36 ± 0.21	0.72
IOL power (D)	20.44 ± 1.68	20.89 ± 2.23	20.03 ± 1.37	0.03*

IOL = intraocular lens, EDoF = extended depth of field, D = diopter.

*Statistically significant difference.

**Fig 1:**
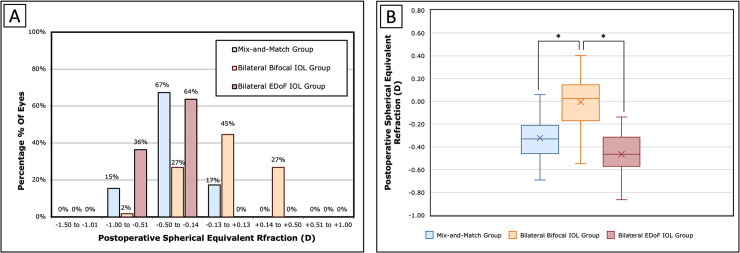
Postoperative spherical equivalent refraction in three groups: the mixed and matched group, the bilateral bifocal IOL group, and the bilateral EDoF IOL group. (A) Distribution of postoperative spherical equivalent refraction across the three groups; (B) Comparison of the mean postoperative spherical equivalent refraction among the three groups. *IOL* = intraocular lens; *EDoF* = extended depth of field; *D* = diopter.

Mean postoperative spherical equivalent was −0.32 ± 0.28 D in the mix-and-match group, with 85% of eyes within ± 0.5D and 100% of eyes were within ± 1.0 D. In the bilateral bifocal IOL group, mean spherical equivalent was 0.01 ± 0.32 D and 98% of eyes were within ± 0.5D and 100% of eyes within ± 1.0 D. In the bilateral EDoF IOL group, mean spherical equivalent was −0.47 ± 0.28 D, with 64% of eyes within ± 0.5D and 100% of eyes within ± 1.0 D (Fig 1A). The mean spherical equivalent in the bilateral bifocal IOL group was significantly higher than the other two groups (both P < 0.01) (Fig 1B).

**Fig 2:**
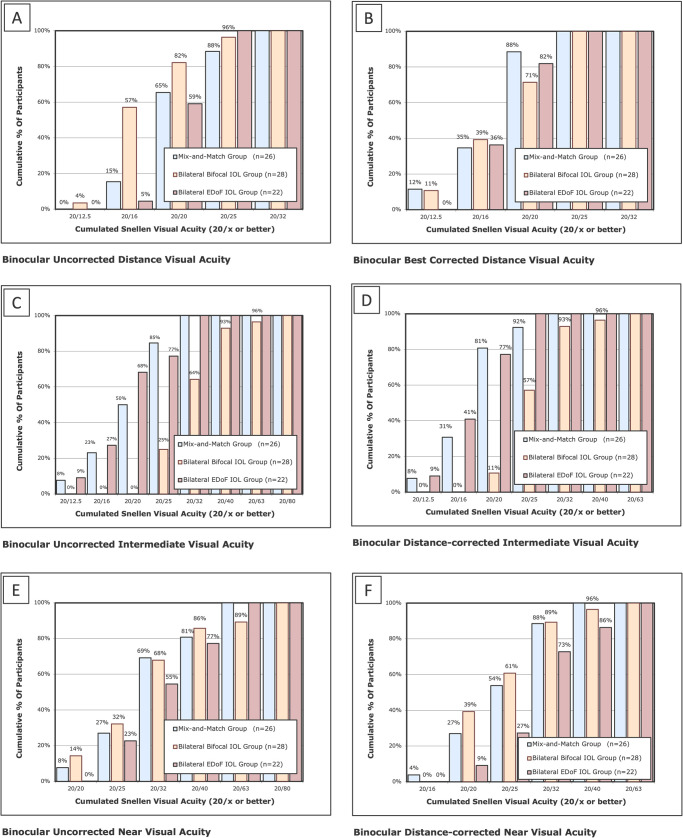
Postoperative binocular visual acuities in three groups: the mixed and matched group, the bilateral bifocal IOL group, and the bilateral EDoF IOL group. (A) Binocular uncorrected distance visual acuity; (B) Binocular best corrected distance visual acuity; (C) Binocular uncorrected intermediate visual acuity; (D) Binocular distance-corrected intermediate visual acuity; (E) Binocular uncorrected near visual acuity; (F) Binocular distance-corrected near visual acuity. *IOL* = intraocular lens; *EDoF* = extended depth of field.

Table 2 summarizes the postoperative mean binocular visual acuities of three groups. All patients achieved UDVA and BCDVA of 20/32 or better in all three groups. In the mix-and-match group and bilateral EDoF IOL group, all patients achieved uncorrected intermediate visual acuity (UIVA) and distance-corrected intermediate visual acuity (DCIVA) of 20/32 or better. In the bilateral bifocal IOL group, 64% of patients achieved UIVA of 20/32 or better, and 93% achieved DCIVA of 20/32 or better (Fig 2). The binocular UIVA and DCIVA were better in the mix-and-match group and bilateral EDoF IOL group (both P < 0.01, see Table 2). The patients in the bilateral bifocal IOL and mix-and-match group showed better distance-corrected intermediate visual acuity (DCNVA) (bilateral bifocal IOL group VS bilateral EDoF IOL group: P = 0.01, mix-and-match group vs. bilateral EDoF IOL group: P = 0.03, see Table 2). By contrast, no significant intergroup differences were observed in fixed-distance measurements of UDVA, BCDVA, or uncorrected near visual acuity (UNVA) at 40 cm (all P > 0.05, see Table 2).

**Table 2:** Comparison of postoperative visual outcomes among three groups.

Parameter	Mix-and-Match Group	Bilateral Bifocal IOL Group	Bilateral EDoF IOL Group	P Value
UDVA (5m, LogMAR)	0.036 ± 0.088	0.059 ± 0.101	0.036 ± 0.053	0.500
BCDVA (5m, LogMAR)	−0.027 ± 0.075	−0.013 ± 0.087	−0.011 ± 0.062	0.715
UIVA (66 cm, LogMAR)	0.042 ± 0.114	0.229 ± 0.100	0.028 ± 0.123	<0.001^*^
DCIVA (66 cm, LogMAR)	−0.004 ± 0.095	0.150 ± 0.093	−0.019 ± 0.081	<0.001^*^
UNVA (40 cm, LogMAR)	0.224 ± 0.118	0.219 ± 0.148	0.262 ± 0.126	0.470
DCNVA (40 cm, LogMAR)	0.135 ± 0.112	0.124 ± 0.130	0.213 ± 0.111	0.020^*^
Spherical equivalent (D)	−0.32 ± 0.28	0.01 ± 0.32	−0.47 ± 0.28	<0.001*

IOL = intraocular lens, EDoF = extended depth of field, UDVA = uncorrected distance visual acuity, BCDVA = best corrected distance visual acuity, UIVA = uncorrected intermediate visual acuity, DCIVA = distance-corrected intermediate visual acuity, UNVA = uncorrected near visual acuity, DCNVA = distance-corrected near visual acuity.

*Statistically significant difference.

**Fig 3:**
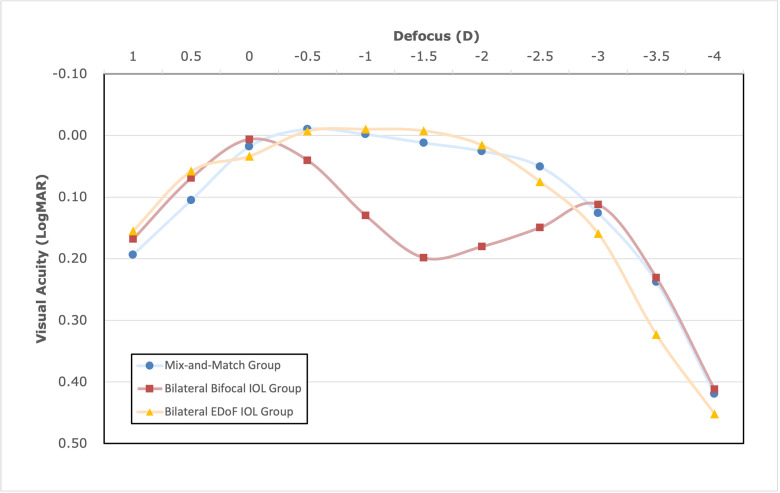
Binocular defocus curves for three groups: the mixed and matched group, the bilateral bifocal IOL group, and the bilateral EDoF IOL group. *IOL* = intraocular lens; *EDoF* = extended depth of field; *D* = diopter.

Binocular defocus curve analysis further characterized visual performance across different defocus levels (Fig 3). The binocular defocus curve of the bilateral bifocal IOL group showed a visual acuity of 0.2 LogMAR or better from −3.0 D to −2.0 D and −1.0 D to +1.0 D. The mix-and-match and bilateral EDoF IOL groups had better visual acuities from −1.0 D to −2.0 D than the bilateral bifocal IOL group (all P < 0.05).

Table 3 shows the responses to the Chinese Cat-Quest 9-SF questionnaire across the three groups. High general satisfaction (very satisfied) rates were reported (86.4% in the bilateral EDoF IOL group, 89.3% in the bilateral bifocal IOL group, and 84.6% in the mix-and-match group), with most patients reporting no difficulty in everyday life (90.9%, 89.3%, and 92.3% in the bilateral EDoF IOL, bilateral bifocal IOL, and mix-and-match groups, respectively). There were no statistically significant differences among the groups in responses to the questionnaire.

**Table 3:** The Chinese Catquest-9SF results.

Question No.	Number (%)			P Value
	Mix-and-Match Group	Bilateral Bifocal IOL Group	Bilateral EDoF IOL Group	
1. Vision satisfaction in general				0.727
Very dissatisfied	0 (0%)	0 (0%)	0 (0%)	
Fairly dissatisfied	3 (11.5%)	1 (3.6%)	1 (4.5%)	
Fairly satisfied	1 (3.8%)	2 (7.1%)	2 (9.1%)	
Very satisfied	22 (84.6%)	25 (89.3%)	19 (86.4%)	
2. Vision difficulty in everyday life				0.592
Very great difficulty	0 (0%)	0 (0%)	0 (0%)	
Great difficulty	1 (3.8%)	0 (0%)	0 (0%)	
Some difficulty	1 (3.8%)	3 (10.7%)	2 (9.1%)	
No difficulty	24 (92.3%)	25 (89.3%)	20 (90.9%)	
3. Reading text in the newspaper				0.391
Very great difficulty	0 (0%)	0 (0%)	0 (0%)	
Great difficulty	0 (0%)	0 (0%)	0 (0%)	
Some difficulty	2 (7.7%)	1 (3.6%)	0 (0%)	
No difficulty	24 (92.3%)	27 (96.4%)	22 (100%)	
4. Recognizing the faces of people around you				0.377
Very great difficulty	0 (0%)	0 (0%)	0 (0%)	
Great difficulty	0 (0%)	0 (0%)	0 (0%)	
Some difficulty	1 (3.8%)	0 (0%)	0 (0%)	
No difficulty	25 (96.2%)	28 (100%)	22 (100%)	
5. Seeing prices of goods when shopping, or descriptions on medicine bottles or bank receipts, electricity bill, water account, etc.				0.576
Very great difficulty	0 (0%)	0 (0%)	0 (0%)	
Great difficulty	1 (3.8%)	0 (0%)	1 (4.5%)	
Some difficulty	3 (11.5%)	1 (3.6%)	1 (4.5%)	
No difficulty	22 (84.6%)	27 (96.4%)	20 (90.9%)	
6. Seeing to walk on uneven ground				
Very great difficulty	0 (0%)	0 (0%)	0 (0%)	
Great difficulty	0 (0%)	0 (0%)	0 (0%)	
Some difficulty	0 (0%)	0 (0%)	0 (0%)	
No difficulty	26 (100%)	28 (100%)	22 (100%)	
7. Reading text on TV or in movie or on advertising board				0.413
Very great difficulty	0 (0%)	0 (0%)	0 (0%)	
Great difficulty	1 (3.8%)	0 (0%)	0 (0%)	
Some difficulty	1 (3.8%)	0 (0%)	0 (0%)	
No difficulty	24 (92.3%)	28 (100%)	22 (100%)	
8. Seeing to do delicate work (needlework, handiwork, carpentry, etc.)				0.863
Very great difficulty	0 (0%)	0 (0%)	0 (0%)	
Great difficulty	1 (3.8%)	0 (0%)	1 (4.5%)	
Some difficulty	3 (11.5%)	3 (10.7%)	2 (9.0%)	
No difficulty	22 (84.6%)	25 (89.3%)	19 (86.5%)	
9. Seeing to carry on an activity/hobby you are interested in, such as photography, calligraphy, Mah-jongg playing				0.543
Very great difficulty	0 (0%)	0 (0%)	0 (0%)	
Great difficulty	0 (0%)	0 (0%)	0 (0%)	
Some difficulty	1 (3.8%)	0 (0%)	1 (4.5%)	
No difficulty	25 (96.2%)	28 (100%)	21 (95.5%)	

IOL = intraocular lens, EDoF = extended depth of field.

**Fig 4:**
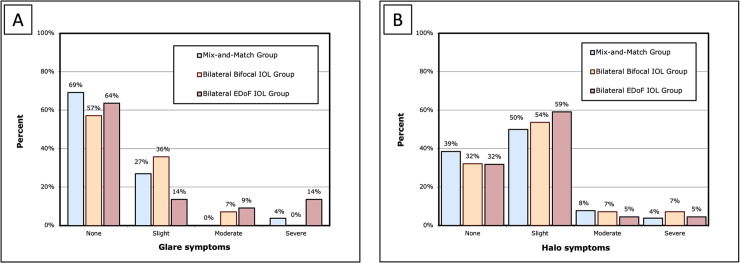
Severity of photic phenomena in three groups: the mixed and matched group, the bilateral bifocal IOL group, and the bilateral EDoF IOL group. (A) Glare symptoms; (B) Halo symptoms. I*OL* = intraocular lens; *EDoF* = extended depth of field.

Fig 4 presents the results regarding glare and halo symptoms. Glare symptoms were perceived by 31% in the mix-and-match group, 43% in the bilateral bifocal IOL group, and 36% in the bilateral EDoF IOL group. Halo symptoms were reported by 61% in the mix-and-match group, 68% in the bilateral bifocal IOL group, and 68% in the bilateral EDoF IOL group. There was no significant difference in the severity of glare and halo symptoms among the three groups (P > 0.05).

Table 4 presents the stereoacuity results. The mean near stereoacuity was 124.09 ± 120.26 arc sec in the bilateral EDoF IOL group, 105.00 ± 78.20 arc sec in the bilateral bifocal IOL group, and 182.69 ± 239.39 arc sec in the mix-and-match group. No statistically significant differences were found in the mean stereoacuity among the three groups. Regarding the proportion of participants achieving stereoacuity of 100 arcsec or better and 40 arcsec or better, the mix-and-match group showed a lower percentage compared with the bilateral EDoF IOL and bilateral bifocal IOL groups, though the differences were not statistically significant.

**Table 4:** Mean stereoacuity and number (percentage) of patients who achieved a disparity threshold of 100 or 40 arcsec or better.

Parameter	Mix-and-Match Group	Bilateral Bifocal IOL Group	Bilateral EDoF IOL Group	P Value
Mean stereoacuity (arc sec)	182.69 ± 239.39	105.00 ± 78.20	124.09 ± 120.26	0.749
Number (%) of patients				
100 arcsec or better	15 (57.69%)	18 (64.29%)	15 (68.18%)	0.775
40 arcsec or better	6 (23.08%)	7 (25.00%)	7 (31.82%)	0.745

IOL = intraocular lens, EDoF = extended depth of field.

## Discussion

The development of society and the improvement in living standards shape people’s visual habits [2]. In recent years, the rise of a cashless society in China has significantly increased the reliance on mobile phones for the Chinese population, including those undergoing cataract surgery, thereby increasing the demand for near vision [9–11]. However, Full vision range IOLs such as trifocal IOL, remain inaccessible in large areas of China, and bifocal and EDoF IOL maintain widespread utilization. To better understand whether the different cataract surgical strategies involving these IOL types would meet the visual needs of the Chinese population, it is of great importance to evaluate the visual outcomes and quality of life involving these cataract surgical methods.

In our study, all three groups achieved good and comparable UDVA and BCDVA. However, the bilateral bifocal IOL group showed lower magnitudes of UIVA and DCIVA compared to the other two groups, and the defocus curve of this group also demonstrated a lower magnitude from a defocus of −1.0 D to −2.0 D. Despite this, the UIVA and DCIVA did not manifest in subjective vision-related quality of life, where the bilateral bifocal IOL group scored favorably across all parameters. Additionally, this group reported the highest overall visual satisfaction and satisfactory daily visual ability (89.3% reported no difficulty), though no significant differences were found among three groups. In this study, the mean UIVA of the bilateral bifocal IOL group was 0.229 logMAR. This result is consistent with the study by John S. M. Chang, which reported similar visual acuity and satisfactory visual quality for this group [5]. Therefore, we believe that the bilateral bifocal IOL implantation strategy, by providing excellent near and distance visual acuity, can offer good quality of life for cataract patients in contemporary Chinese society. We speculate that a UIVA level of 0.229 is generally sufficient for tasks requiring intermediate vision, such as cooking, playing cards, and face-to-face conversations, as they typically do not require high visual acuity for perceiving fine details. Additionally, patients can manually adjust their viewing distance to accommodate further visual needs. However, it is important to note that this study was a non-randomized, real-world study, and patients selected their implantation strategy based on personal preference. The patients in this group were more likely to have higher demands for near vision. Furthermore, they were informed in advance about the potential limitations in intermediate visual acuity, which could explain their high satisfaction levels despite this issue. Nevertheless, this non-randomized design may better reflect the real-world clinical outcomes of IOL implantation aimed at meeting patient needs compared to randomized trials.

In this study, the bilateral EDoF IOL group provided good corrected and uncorrected visual acuity from distance to intermediate ranges. The defocus curve for this group showed stable and favorable visual acuity values from −1.0D to −2.5D, but a continuous decline from −2.5D to −4.0D. Although the UNVA in this group was lower than that in the other two groups, the difference was not statistically significant. This may be attributed to the surgeons’ preference for selecting negative diopters when determining the target refractive power for the TECNIS Symfony ZXR00 IOL. Additionally, according to the questionnaire results, 100% of the patients in this group reported that they were able to read newspapers and clearly see text on mobile phones with no difficulty. A similar surprising outcome was also reported in the study by Oh-sub Koo et al [8]. In their study, the group with bilateral EDoF IOL implantation achieved the highest rate of spectacle independence for near vision, compared to the other two groups using the mix-and-match technique with two different bifocal IOLs [8]. Given that a visual acuity of 30/60 is sufficient for newspaper reading; the mean UNVA for this group is 0.262, greater than the level of 30/60 [20]. Thus, the near vision provided by this group may be adequate to meet the daily demands of mobile phone use and other near-vision tasks.

In our study, the mix-and-match group exhibited the broadest range of the defocus curve, with strong performance across the defocus range from 0 to −3.0 D. Additionally, the group demonstrated excellent visual acuity for distance, intermediate, and near ranges. In the studies on the mix-and-match IOL approach, most bifocal IOLs used had a + 3.5 D near addition, and to the best of our knowledge, only one study has reported the visual outcomes for mix-and-match approach with +4.0 D near addition bifocal IOL [8]. In this study, we used a bifocal IOL with a + 4.0D near addition for mix-and-match approach and presented excellent visual outcomes and vision-related quality of life. Compared with the other two groups, it had the broadest defocus curve, which is the greatest advantage of this approach.

There are some limitations in this study. First, the study was not a randomized design, the participants selected IOL based on their own preference. To minimize patient bias, randomized group assignment is considered the most appropriate. However, it is important to both the patients and us that the participants can receive an IOL that meets their visual demands. Besides, patient involvement in the selection of IOL may better reflect real-world clinical conditions. Second, this study is a single-center study conducted at a tertiary hospital. As a result, the patient population may consist of individuals with higher education levels, whose demands for near and intermediate vision may not fully represent the characteristics of the overall Chinese population. Therefore, a multi-center study involving a more diverse population is needed for further investigation. Third, trifocal and EDoF multifocal IOL were not included in this study. The use of these types of IOLs may come close to achieving the goal of all-distance vision. However, these lenses are not widely used or popular, as they are inaccessible in most areas of China and their cost remains prohibitively high for most patients.

In conclusion, all three groups achieved good vision-related quality of life, with a superiority of the mix-and-match and bilateral EDoF IOL groups in intermediate vision and better bilateral visual outcomes in near distance in the mix-and-match and bilateral bifocal IOL groups. These approaches remain valuable alternatives for providing optimal visual outcomes in regions where full vision range IOL are not yet accessible.

## Supporting information

S1 Appendix Preoperative lifestyle and visual needs questionnaire (English translation). (PDF)

S2 Appendix Photic phenomena questionnaire (English translation). (PDF)
