# Supplementary material for: Visual outcomes and quality of life after bilateral extended depth of field, bifocal, and mix-and-match IOL implantation
Source: PLoS One. 2026 Feb 6;21(2):e0341136. doi: 10.1371/journal.pone.0341136 (PMC12880696; doi:10.1371/journal.pone.0341136)
Supplement: S1 Appendix — (PDF) [file pone.0341136.s001.pdf]

Preoperative Questionnaire (English Version)

Part I (Completed by Surgeon)

1. Are there any ocular diseases that may affect visual acuity (e.g., retinal diseases, corneal diseases)?

☐ Yes, please specify: ____________ ☐ No

2. Are there any systemic diseases that may affect visual prognosis (e.g., diabetes mellitus, psychiatric disorders)?

☐ Yes, please specify: ____________ ☐ No

3. Do the biometric results potentially affect the performance of functional intraocular lenses (e.g., kappa angle > 0.5 mm, total higher-order aberrations > 0.5 µm, irregular astigmatism)?

☐ Yes, please specify: ____________ ☐ No

If all answers above are "No," proceed to Part II.

Part II (Completed by Patient)

1. What is the greatest inconvenience caused by your visual problems in daily life?

____

____

2. After your vision is restored, what is the thing you most want to do?

____

____

3. Based on your family's financial situation, what is the maximum acceptable cost of surgery (per eye)?

☐ < 5,000 RMB ☐ 5,000–7,000 RMB ☐ 7,000–10,000 RMB ☐ > 10,000 RMB

4. Do you have high myopia (> –6.00 D / > 600 degrees)?

☐ Yes — Can you accept the need to continue wearing myopic glasses after surgery?

☐ Acceptable ☐ Barely acceptable ☐ Unacceptable ☐ Do not mind

☐ No — Can you accept the need to wear reading glasses for near vision after surgery (e.g., using a mobile phone, reading newspapers)?

☐ Acceptable ☐ Barely acceptable ☐ Unacceptable ☐ Do not mind

For each activity below, how often does it occur in your daily life?

☐ Daily ☐ Often ☐ Sometimes ☐ Rarely

Distance Vision

Activity	Daily	Often	Sometimes	Rarely
Recognizing acquaintances approaching	☐	☐	☐	☐
Walking on stairs, steps, or uneven surfaces	☐	☐	☐	☐
Watching television	☐	☐	☐	☐

Intermediate Vision

Activity	Daily	Often	Sometimes	Rarely
Using a computer	☐	☐	☐	☐
Playing mahjong, cards, chess, or similar board/card games	☐	☐	☐	☐
Cooking or preparing meals	☐	☐	☐	☐

Near Vision

Activity	Daily	Often	Sometimes	Rarely
Using a mobile phone; reading; writing	☐	☐	☐	☐
Reading small print (e.g., medicine labels, product labels, receipts, a watch)	☐	☐	☐	☐
Fine handwork (e.g., threading a needle, sewing, knitting)	☐	☐	☐	☐

Moving Objects

Activity	Daily	Often	Sometimes	Rarely
Sports/activities (e.g., table tennis, badminton, square dancing)	☐	☐	☐	☐
Driving during the daytime	☐	☐	☐	☐
Driving at night	☐	☐	☐	☐

Personality Rating

Please rate your personality using the scale below:

0 = very easygoing; 10 = perfectionist

0 ———————————————————————— 10

Very easygoing | Perfectionist
