# Supplementary material for: Visual outcomes and quality of life after bilateral extended depth of field, bifocal, and mix-and-match IOL implantation
Source: PLoS One. 2026 Feb 6;21(2):e0341136. doi: 10.1371/journal.pone.0341136 (PMC12880696; doi:10.1371/journal.pone.0341136)
Supplement: S2 Appendix — (PDF) [file pone.0341136.s002.pdf]

Photic Phenomena Questionnaire

(English translation)

For patient completion

Do you experience glare in your daily life?

Please rate its severity on a scale from 0 to 5

0 none 5 very severe

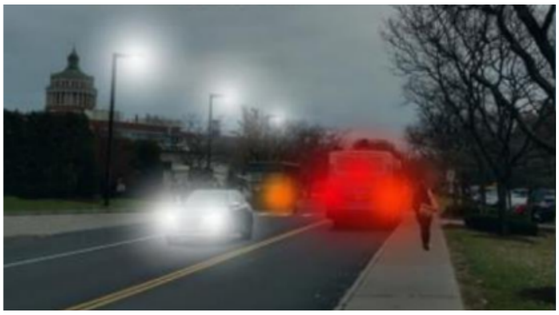

Do you experience halo in your daily life?

Please rate its severity on a scale from 0 to 5

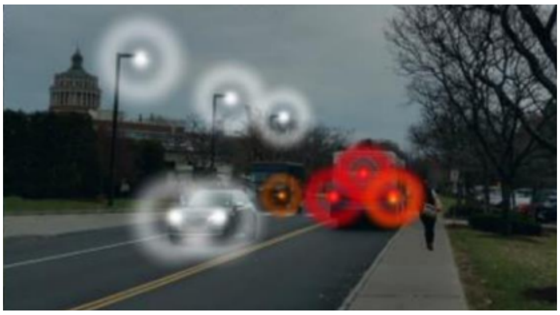

0 none 5 very severe
